# Matching Is Best: Enhancing Effects of Future Orientation and Construal Level on Green Consumption

**DOI:** 10.3390/bs14111100

**Published:** 2024-11-15

**Authors:** Yidi Chen, Qinxin Liu, Shuyu Shan, Cancan Jin

**Affiliations:** School of Humanities and Social Sciences, Beijing Forestry University, Beijing 100083, China; 200802209@bjfu.edu.cn (Q.L.); ssy3230804@bjfu.edu.cn (S.S.); jincancan@bjfu.edu.cn (C.J.)

**Keywords:** future orientation, green consumption, construal level, enhancement effect, moderation model

## Abstract

The 2024 Report on the Work of the Chinese Government promotes trade in consumer goods and green consumption. Therefore, better understanding is needed of consumer attitudes and behaviors toward environmental protection and sustainable development. The relationships among future orientation, construal level, and green consumption were explored using a delayed-effect design to conduct two surveys at one-month intervals with 160 participants (100 women and 60 men). The results showed that (1) future orientation positively predicted green consumption intention and green consumption behavior, and (2) the construal level significantly moderated the influence of future orientation on green consumption. Specifically, a high construal level and high future orientation predicted the greatest green consumption intention, green consumption behavior, and green consumption decisions, while at a high construal level, future orientation positively predicted green consumption intention and behavior, with a greater tendency to purchase environmentally friendly rather than hedonic products. At a low construal level, future orientation had no significant effect on green consumption intention, behavior, and intention to purchase environmentally friendly products. This study is important for promoting public awareness of the importance of green consumption and encouraging green consumption behaviors to achieve the goals of sustainable development and protect the environment and social well-being.

## 1. Introduction

With the acceleration of industrialization, urbanization, and other processes, environmental problems have become an important challenge for the humankind [1]. With the increasing severity of global environmental problems, sustainable development has become an inevitable choice for the human society. Better understanding of consumer attitudes and behaviors toward environmental protection and sustainable development is urgently needed [2].

Green energy consumption is an important means for achieving sustainable development. It emphasizes saving resources, reducing pollution, and protecting the environment, which helps maintain ecological balance and meet people’s ecological needs. Green consumption can reduce damage to the environment, promote the harmonious coexistence of humans and nature, and lay the foundation for sustainable development [3]. Therefore, it is particularly important to explore the mechanisms underlying the influence of green consumption. Green consumption refers to environmentally friendly consumption behaviors. Typical green consumption behaviors include purchasing environmentally friendly items, such as recycled paper, second-hand products, and energy-saving appliances, as well as saving water and electricity [4].

Green consumption is a process that is strongly influenced by consumer values, norms, and habits and is also a very complex, diverse, and context-dependent process [5]. In addition to the characteristics of the product itself (e.g., packaging, colors, etc.) [6,7,8], much of the previous research has explored the influence of factors such as environmental values and environmental knowledge in promoting green consumption behavior [9,10,11]. There are also studies that explore how policies influence green consumption [1,12]. However, policies often take a long time to develop, and, thus, looking at individual factors to change green consumption can provide good insights for interventions [13]. Environmental values and environmental concerns, both concepts often used to predict green consumption, are associated with an individual’s assessment that present behavior affects the future [14,15]. And considering green consumption is an oxymoron, which implies the protection of the environment and resources that are eco-friendly but are usually more expensive than ordinary products [5]. Therefore, more cognitive resources are required to promote green consumption. Thus, time perspective is an important personal factor influencing green consumption.

However, there is a gap between intentions and behaviors in the area of green consumption. That is, most people can realize the benefits of green consumption and have the intention to consume in this way; however, there is still a gap between green consumption intentions and green consumption behaviors [16]. Therefore, relying only on self-reports of green consumption intention does not provide a comprehensive measure of an individual’s green consumption behavior. Meanwhile, although recent publications have demonstrated the validity of self-report scales [17], the use of multiple metrics to assess variables together and to be able to obtain consistent results is important in addressing the crisis of reproducibility in psychological research. Therefore, with reference to the indicators assessed in a previous study [18], in addition to the self-report scale, this study also introduced an actual behavioral decision-making paradigm for the purchase of environmentally friendly products in order to improve the validity of research in the pro-environmental domain [19].

### 1.1. Future Orientation to Promote Green Consumption

Temporal perspective theory suggests that behaviors and decisions are influenced by different temporal cognitive processes [20], which are primarily classified into future orientation, past orientation, and present orientation, according to the point of time of concern. Future orientation implies that people pay attention to future developments and changes and prepare for them accordingly, including the importance of future life domains, positive evaluations of future life, high expectations of success, and positive emotions [21]. Because of having a long-term orientation framework and more positive future expectations [22], individuals with a strong future orientation tend to have more cognitive resources and stronger self-control [23]. Numerous studies have demonstrated a positive association between future orientation and environmental behavior [24,25,26]. At the individual level, individuals with high future orientation engage in more environmentally friendly behaviors, e.g., future orientation is significantly positively correlated with household environmental attitudes and significantly negatively correlated with energy consumption [24]; it has also been demonstrated that the planning dimension of future orientation is significantly positively correlated with green consumption among individuals from different cultures [25]. At the national level, citizens who use strong future-tense language are more concerned about environmental issues related to climate change and produce less carbon emissions and energy waste [26]. This is because, from one perspective, green consumption, as a type of environmental behavior, has particularly significant long-term benefits; therefore, individuals are more likely to choose green consumption when they are aware that such behavior will have a positive impact on the future environment. In contrast, green consumption encompasses more innovative elements than traditional consumption, and individuals with a strong future orientation are more likely to accept innovative thinking [4]. Therefore, future orientation and green consumption are positively correlated in many contexts.

However, studies also show no or a negative correlation between future orientation and pro-environmental behaviors, such as green consumption [18,27,28]. Bruder et al. found no direct correlation between delayed discounting, which characterizes future orientation, and green consumption [27]. The relationship between nostalgia, the counterpart of future orientation, and green consumption has also been explored by many researchers. It has been argued that nostalgia, which represents a past orientation, reduces green consumption. Khan et al. (2021) argued that nostalgia stimulates green consumption. This is primarily because nostalgia stimulates an individual’s sense of collective efficacy and, therefore, increases the promotion of social norms toward environmentally friendly behavior [18,28]. These studies challenge the notion of a stable positive relationship between future orientation and green consumption and suggest that future orientation requires the addition of marginal conditions to facilitate green consumption. However, future orientation, as a temporal perspective, needs to be combined with a framework to explain individual decisions and behaviors [29].

### 1.2. The Moderating Role of the Level of Explanation

The construal level framework can be used in conjunction with the temporal orientation framework to explain the occurrence of green consumption [30]. The construal level refers to the degree to which an individual abstracts or represents an objective entity [31]. Construal level theory suggests that individuals with high construal levels tend to focus on the holistic perspective of the object, focusing on value rather than feasibility [32]. For example, they may consider the consequences to be brought about by themselves. In contrast, individuals with a low construal level tend to focus on the local perspective of the object, focusing on feasibility [33]. That is, they tend to ignore the environmental factors underlying products.

The augmentation effect of temporal fit suggests that the combination of the construal level and temporal perspective can explain individual green consumption choices [34]. Specifically, a focus on ‘concrete’ and ‘contextualization’ is more likely to contribute to low levels of construal. When individuals are more concerned with low levels of construal, even though individuals have higher future orientations, they are prone to be preoccupied with immediate issues due to a focus on information that is more contextualized and specific, and they easily ignore the long-term outcome of the problem and, thus, are not inclined to choose green consumption that is more favorable to the environment [35]. In contrast, attention to the ‘abstract’ is more likely to contribute to high levels of construal [36]. When individuals have high levels of construal, they can abstract from individual factors, and if individuals have a longer-term temporal orientation at this point in time, the future orientation will work better while focusing on the macro-consequences, thus contributing to green consumption.

### 1.3. The Present Study

Among the facilitators of green consumption, previous studies have primarily considered future orientation as an antecedent variable for promoting green consumption at the individual level [25,26]. However, previous studies have shown inconsistencies in the relationship between future orientation and green consumption [16,28]. This may be due to the low ecological validity of measures of green consumption [19]. Second, moderating variables exist concerning the effect of future orientation on green consumption. Time perspective theory suggests that future orientation can promote individuals’ green consumption [20]; however, it cannot explain the positive relationship between past orientation and green consumption [32,34]. However, construal-level theory and the augmentation effect of time fit suggest that the construal level can play a moderating role in the effect of future orientation on green consumption. Therefore, this study explored green consumption from the perspective of future orientation and investigated the moderating role of the construal level. At the same time, previous studies have mostly explored the relationship between future orientation and green consumption in terms of self-report scales, which limits the ecological validity of this area of research. The product choice paradigm has been widely used in research in the field of consumption [37]. Previous studies presented figures of products with environmental and hedonic attributes to subjects, and the participants’ choices reflected individuals’ green consumption decisions. Products with environmental attributes are those that are more environmentally friendly and greener, while hedonistic products are those that are more comfortable and make people happy [38].

To enhance the validity of the research in this area, this study conducted a more comprehensive assessment of green consumption, including not only green consumption intentions and green consumption behaviors but also actual purchasing decisions for environmentally friendly and non-environmentally friendly attributes of products to make this study more practically valuable.

The following hypotheses were proposed:

**Hypothesis 1.**  *Future orientation affects green consumption and not only positively predicts green consumption intentions and self-reported behaviors but also positively predicts green consumption decision. The higher the future orientation, the more inclined the individual is to consume environmental products rather than hedonic products.*

**Figure 1:**
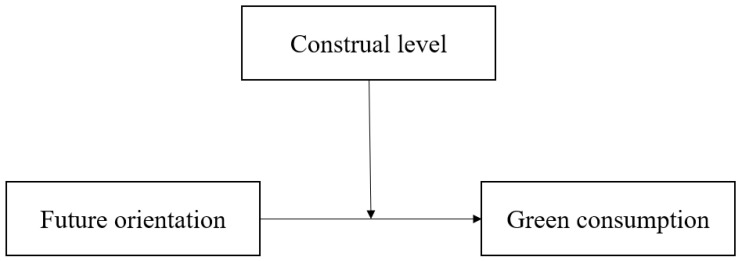
Hypothesized model.

**Hypothesis 2.**  *The construal level can moderate the influence of future orientation on green consumption; specifically, in a high construal level group, future orientation has a stronger influence on green consumption, while in a low construal level group, no such relationship is present (see Figure 1).*

## 2. Methods

### 2.1. Research Design and Process

This study used a questionnaire and convenience sampling to recruit participants. The questionnaire was a web-based questionnaire, created using the Questionnaire Star platform (https://www.wjx.cn/, accessed on 18 January 2024), which is a very useful online survey instrument in China. The questionnaire was applied twice at an interval of 1 month. Participants were recruited online through posters. The two questionnaires were matched using the unique id of the participants. This study was conducted in stages, with informed consent collected from the participants beforehand. In the first stage of this study, 196 participants completed a group test in January 2024 (hereafter referred to as T1) to determine their consideration of future results, construal level, and demographic information, which took approximately 5 min. The second stage of this study was conducted after a 1-month interval (hereinafter referred to as T2). Participants who completed the first questionnaire were invited to complete a questionnaire about green consumption and purchase decisions for products with different attributes in a collective manner at T2. Data from 169 participants were collected, and 17 samples were lost (13.78%). An attention screening question (“Please select 2 for this item”) was used to eliminate questionnaires of inattentive individuals. The results of the two questionnaires were then matched. A total of 160 valid questionnaires were collected for an effective sample recovery rate of 86.22%. The participants consisted of 100 women (62.50%) and 60 men (37.50%). The mean age of the participants was 22.56 years (SD = 6.03). This study received ethical review at the first author’s site, and all participants volunteered to participate. After completing all research tasks, each participant was paid RMB 5.

### 2.2. Research Tools

#### 2.2.1. Future Orientation

Future orientation was measured using the Consideration of Future Consequences—14 Scale (CFC-14). This scale was adapted from the Consideration of Future Consequences Questionnaire of Joireman [39]. The scale is divided into two dimensions: present and future. Questions 1, 2, 6, 7, 8, 13, and 14 assess the future dimension. An example item is, “I will consider the situation of things in the future and try to influence these things through daily behaviors”. Questions 3, 4, 5, 9, 10, 11, and 12 assess the present dimension. An example item is, “whether convenience is an important factor affecting my decision or action”. The scale collects responses using a 7-point Likert-type scale ranging from 1 to 7 (“strongly disagree” to “strongly agree”). Researchers have proposed that the degree of future orientation can be measured as the sum of the inverse of the present dimension inversion and the future dimension [40]. Therefore, in this study, future orientation was obtained in this manner. The Cronbach’s α value for the scale in the current study was 0.71.

#### 2.2.2. Construal Level

The construal level was measured using the Behavior Identification Form (BIF) compiled by Vallacher and Wegner based on action recognition theory [41]. There are 25 options that are scored as 1 and 0, with 1 representing abstract construal and 0 representing concrete construal. For example, one item asks whether making a list represents “organizing activities” (1 point) or “taking detailed notes” (0 points). The lower the score, the more specific the construal, and vice versa. In this experiment, the Cronbach’s α value of this scale was 0.74.

#### 2.2.3. Green Consumption Scale

The approach of Mamun et al. (2018) was used to measure green consumption intention and behavior as two independent dimensions [42]. Each aspect was assessed via seven items. The willingness to consume green products was assessed by asking the participants about their green consumption intentions (purchase of environmentally friendly products). The green consumption behavior scale uses words that indicate what the respondent has done or is doing, such as using, intentionally avoiding, intending to buy, and so forth. In this experiment, Cronbach’s α values for green consumption intention and green consumption behavior were 0.85 and 0.82, respectively.

#### 2.2.4. Purchase Decisions for Products with Different Attributes

**Figure 2:**
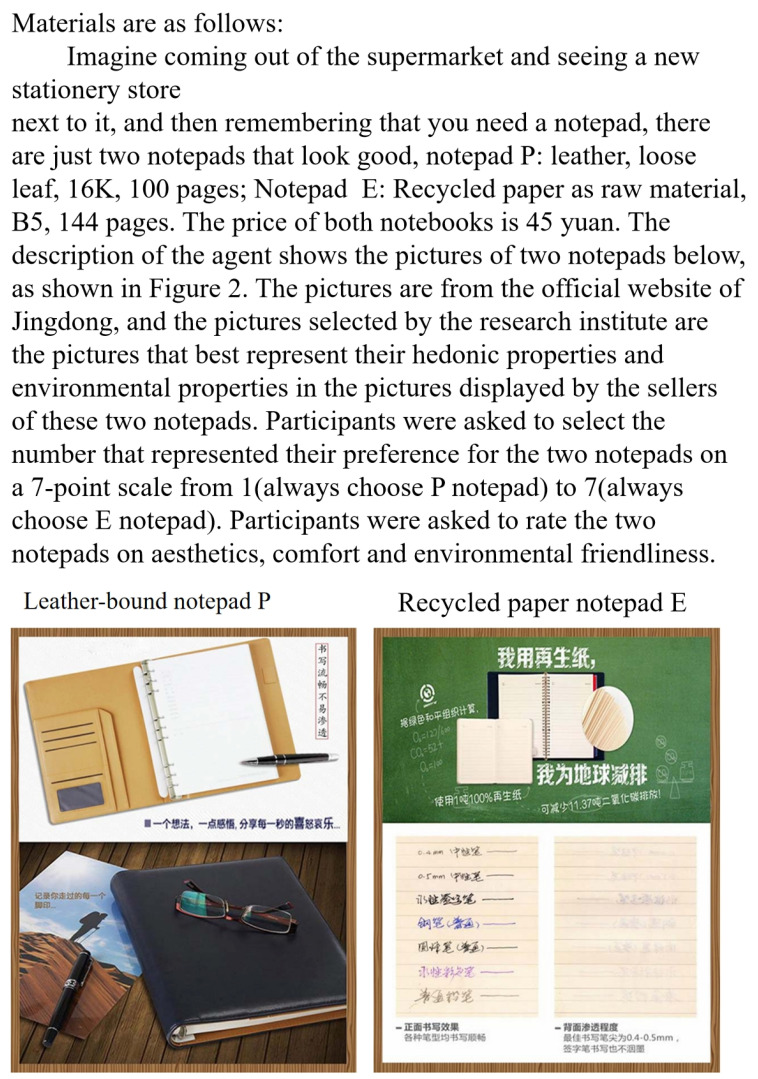
Images of products with different attributes.

The materials for the purchase decisions of products with different attributes were taken from Wu et al. (2016) [43]. Images were obtained from the official website of Jingdong. The pictures selected for the research were those displayed by the sellers of two notepads that best represented their hedonic and environmental properties. Wu et al. (2016) did a prior rating to the materials, in which they invited the participants to rate the two notebooks for comfort, aesthetics, and environmental friendliness in a 1 to 7 Likert scale and obtained that the hedonistic product notebookP was more comfortable and beautiful than the eco-friendly product notebookE. In terms of environmental friendliness, the eco-friendly product notebookE was more eco-friendly than the hedonistic product notebook (Figure 2).

#### 2.2.5. Data Analysis

SPSS (version 25.0) was used for data entry, sorting, and analyses. First, the Harman single-factor test (SPSS 25.0) was used to test the common method bias of the data at the two time points. Second, SPSS 25.0 was used for the correlation analysis of the major variables. Finally, Model 1 of PROCESS macro was used to test the moderating effects of future orientation, construal level, green consumption, and purchase decisions for products with different attributes. The ggplot function (R 4.0) was used to visualize the data.

## 3. Results

### 3.1. Common Method Bias Test

The Harman single-factor test was used to test the common method bias of the two questionnaires [44]. The results showed that the total number of factors with eigenvalues greater than one was 17, and the variance explained by the first common factor was 15.58%, which was less than the critical value of 40%. This indicates that there was no serious methodological bias in the data used in this study.

### 3.2. Descriptive Statistics and Correlation Analysis

Pearson’s correlation analysis was conducted for future orientation, construal level, green consumption intention, green consumption behavior, and purchase decisions. The results showed that future orientation at T1 was significantly correlated with the construal level at T1 (*r* = 0.25, *p* < 0.01), green consumption intention at T2 (*r* = 0.37, *p* < 0.01), and green consumption behavior at T2 (*r* = 0.36, *p* < 0.01). Purchase decisions were significantly correlated with green consumption intention at T2 (*r* = 0.31, *p* < 0.01) and green consumption behavior at T2 (*r* = 0.39, *p* < 0.01). Future outcome considerations were positively correlated with construal levels and green consumption behavior. The construal level was positively correlated with green consumption behavior. Green consumption behavior was positively correlated with purchase decisions. There was no significant correlation between purchase decisions and future orientation or the construal level (Table 1).

### 3.3. Moderating Effect of Future Orientation, Construal Level, and Green Consumption Intention

To verify the effect of the construal level on the relationship between future orientation and green consumption intention, PROCESS macro Model 1 was used to conduct a moderating effect test. T1 future orientation was taken as the independent variable, the T1 construal level as the moderating variable, and T2 green consumption intention as the dependent variable, adjusting for sex and age. The results of the model test showed that the model fit the data significantly well (*R*^2^ = 0.18, *F*(5,154) = 6.61, *p* < 0.001). Future orientation at T1 positively predicted green consumption intention at T2 (*β* = 0.29, SE = 0.06, *t* = 4.67, *p* < 0.001, 95% CI [0.17, 0.41]). The construal level at T1 did not predict green consumption intention at T2 (*β* = −0.03, SE = 0.06, *t* = −0.57, *p* = 0.572, 95% CI [−0.16, 0.09]). The construal level at T1 significantly moderated the influence of T1 future orientation on T2 green consumption intention (Δ*R*^2^ = 0.03, *β* = 0.15, SE = 0.06, *t* = 2.52, *p* = 0.013, 95% CI [0.03, 0.27]). Sex was not a significant predictor (*β* = 0.01, SE = 0.06, *t* = 0.30, *p* = 0.762, 95% CI [−0.10, 0.14]), but age had a significant positive predictive effect on T2 green consumption behavior (*β* = 0.14, SE = 0.06, *t* = 2.30, *p* = 0.023, 95% CI [0.02, 0.26]).

**Figure 3:**
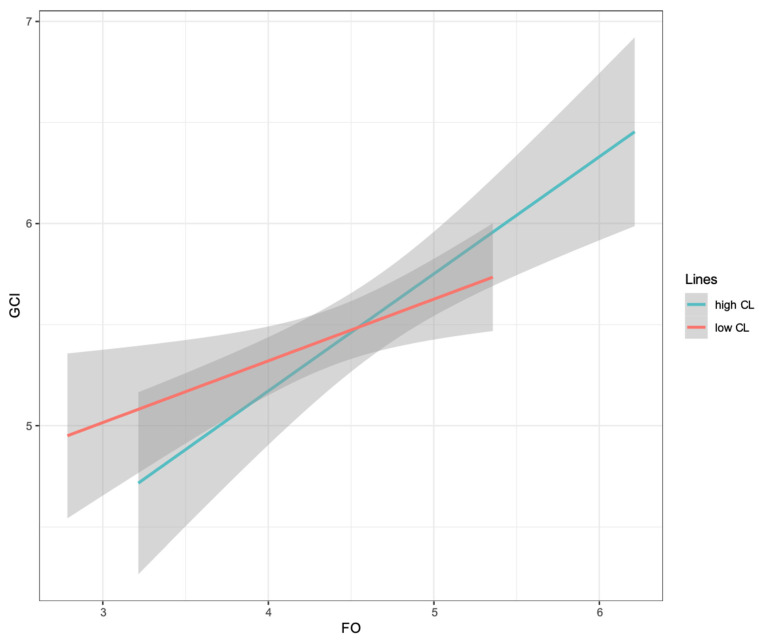
Moderating effect diagram. CL = construal level; FO = future orientation; GCI = green consumption intention.

A simple slopes plot is shown in Figure 3. The results of the simple slopes analysis revealed that future orientation positively predicted green consumption intention when the construal level was high (*β* = 0.44, SE = 0.08, *t* = 5.43, *p* < 0.001, 95% CI [0.28, 0.60]). At low construal levels, future orientation had no significant effect on green consumption intention (*β* = 0.14, SE = 0.09, *t* = 1.53, *p* = 0.129, 95% CI [−0.04, 0.32]).

### 3.4. Future Orientation, Construal Level, and Moderating Effect Test of Green Consumption Behavior

To verify the effect of the construal level on the relationship between future orientation and green consumption behavior, PROCESS macro Model 1 was used to conduct the moderating effect test. T1 future orientation was taken as the independent variable, the T1 construal level as the moderating variable, and T2 green consumption behavior as the dependent variable, adjusting for sex and age. The results of the model test showed that the model fit the data significantly well (*R*^2^ = 0.19, *F*(5,154) = 7.42, *p* < 0.001). T1 future orientation positively predicted T2 green consumption behavior (*β* = 0.26, SE = 0.06, *t* = 4.23, *p* < 0.001, 95% CI [0.14, 0.39]). The T1 construal level did not predict T2 green consumption intention (*β* = 0.04, SE = 0.06, *t* = 0.57, *p* = 0.567, 95% CI [−0.09, 0.16]). The T1 construal level significantly moderated the influence of T1 future orientation on T2 green consumption behavior (Δ*R*^2^ = 0.03, β = 0.15, SE = 0.06, *t* = 2.52, *p* = 0.012, 95% CI [0.03, 0.27]). Sex was not a significant predictor (*β* = 0.01, SE = 0.06, *t* = 0.30, *p* = 0.762, 95% CI [−0.10, 0.14]), although age was a significant positive predictor of T2 green consumption behavior (*β* = 0.14, SE = 0.06, t = 2.30, *p* = 0.023, 95% CI [0.02, 0.26]).

**Figure 4:**
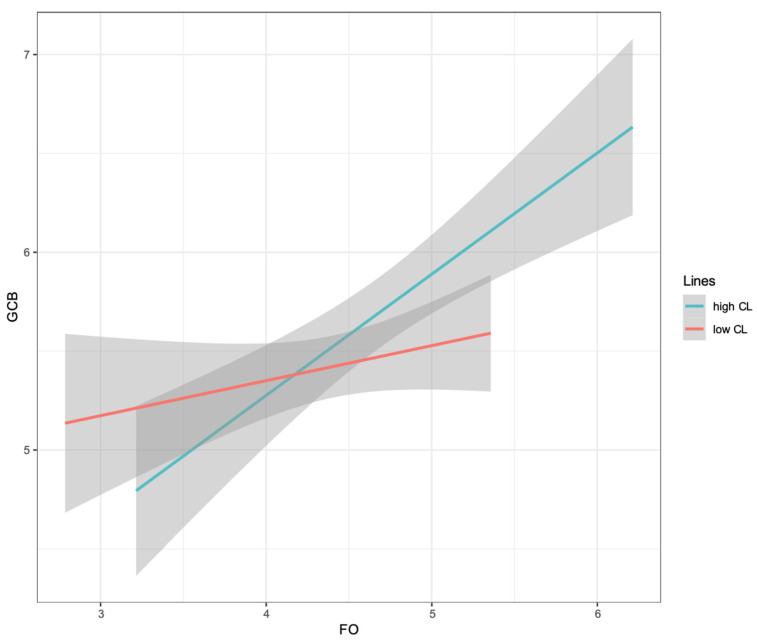
Moderating effect diagram. CL = construal level; FO = future orientation; GCB = green consumption behavior.

A simple slopes plot is shown in Figure 4. The results of the simple slopes analysis found that future orientation positively predicted green consumption behavior when construal levels were high (*β* = 0.41, SE = 0.08, *t* = 5.09, *p* < 0.001, 95% CI [0.25, 0.58]). At low construal levels, the effect of future orientation on green consumption was not significant (*β* = 0.11, SE = 0.09, *t* = 1.22, *p* = 0.223, 95% CI [−0.07, 0.29]).

### 3.5. Moderating Effect Test of Future Orientation, Construal Level, and Purchase Decisions for Products with Different Attributes

PROCESS macro Model 1 was used to verify the effect of future orientation on the relationship between the construal level and the decision to purchase products with different attributes. T1 future orientation was taken as the independent variable, the T1 construal level as the moderating variable, and the purchase decisions for products with different attributes at T2 as the dependent variable. The moderating effect analysis was adjusted for sex and age. The results showed that the model fit the data significantly well (*R*^2^ = 0.07, *F*(5,154) = 2.32, *p* = 0.046). T1 future orientation did not predict the decision to purchase products with different attributes (*β* = 0.10, SE = 0.11, *t* = 0.92, *p* = 0.358, 95% CI [−0.11, 0.31]) nor did the T1 construal level (*β* = −0.01, SE = 0.11, *t* = −0.06, *p* = 0.953, 95% CI [−0.22, 0.21]). The T1 construal level significantly adjusted the influence of T1 future orientation on the decision to purchase products with different attributes (Δ*R*^2^ = 0.05, *β* = 0.29, SE = 0.10, *t* = 2.84, *p* = 0.005, 95%CI [0.09, 0.50]). Sex was not a predictor (*β* = 0.10, SE *=* 0.10, *t* = 1.00, *p* = 0.319, 95% CI [0.10, 0.31]) nor was age (*β* = 0.10, SE = 0.10, *t* = 0.92, *p* = 0.358, 95% CI [0.11, 0.30]).

**Figure 5:**
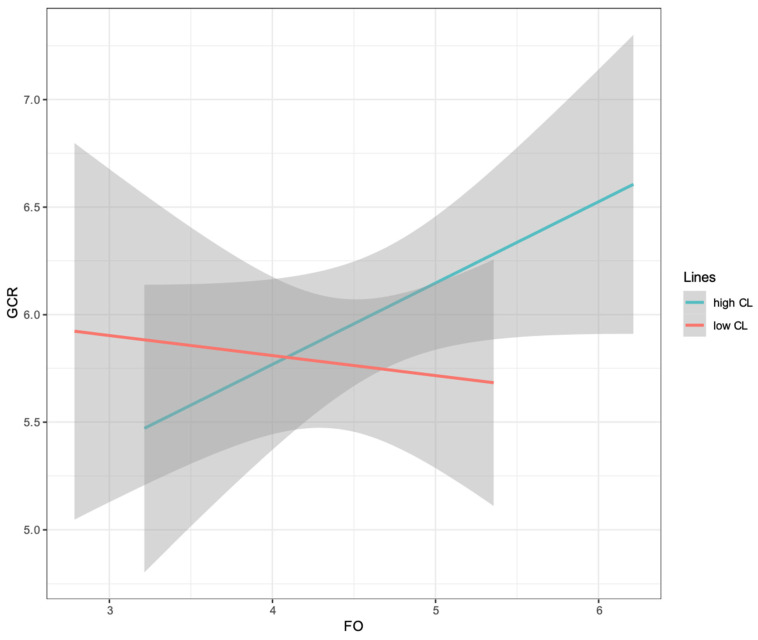
Moderating effect diagram. CL = construal level; FO = future orientation; GCR = intention to purchase products with different attributes.

A simple slopes plot is shown in Figure 5. In the chart, the horizontal coordinate represents future orientation, and the vertical coordinate represents the decision to purchase products with different attributes. The smaller the vertical axis value, the more inclined the respondent is toward hedonic products, whereas the larger the vertical axis value, the more inclined the respondent is toward environmental products. The results of the simple slopes analysis showed that future orientation had a significant impact on green consumption at a high construal level (*β* = 0.39, SE = 0.14, *t* = 2.80, *p* = 0.006, 95% CI [0.12, 0.67]). At a low construal level, the influence of future orientation on the decision to purchase products with different attributes was not significant (*β* = −0.19, SE = 0.16, *t* = −1.24, *p* = 0.327, 95% CI [−0.50, 0.12]).

## 4. Discussion

Through a delayed effect study design with a one-month interval, we found that future orientation can promote individuals’ green consumption intention and green consumption behavior in the short term, partially verifying Hypothesis 1. At the same time, the construal level can modulate the effect of future orientation on green consumption intention and green consumption behavior one month later, as well as on purchase decisions for items with different attributes, verifying Hypothesis 2. Specifically, future orientation can promote individuals’ green consumption behavior only when the construal level was high, demonstrating an enhancement effect of future orientation and the construal level.

### 4.1. Contribution of Future Orientation to Green Consumption

Hypothesis 1 was confirmed, namely individuals with a high future orientation tended to have higher green consumption intentions and green consumption behaviors, which validates time perspective theory [20]. Although previous studies verified a positive relationship between nostalgia and green consumption, nostalgia works by increasing collective efficacy [18,28]. From a time perspective, individuals with longer-term thinking can consider the impact of present actions on future events; therefore, for such individuals, the positive relationship between future orientation, green consumption intention, and green consumption behavior is more stable. At the same time, individuals with a future orientation tend to have more cognitive resources and greater self-control, which makes them more likely to make decisions that are beneficial to the environment rather than to themselves in the present when faced with a scenario that requires cognitive effort [23]. This suggests that we can design future orientation interventions to enhance individuals’ green consumption intentions and promote their green consumption behaviors from a time management perspective. Currently, there are future orientation interventions in the educational [45] and psychotherapeutic domains [46,47], but the application of the intervention in the pro-environmental domain is still very rare. Therefore, this paper provides theoretical guidance for developing future orientation interventions to enhance green consumption.

However, there was no correlation between future orientation and behavioral decision-making for green consumption, which verifies that future planners may, nevertheless, not execute such plans and that there exists separation between cognition and behavior when making environmental purchases [19]. In an actual purchasing behavior scenario, an individual’s cognitive resources are occupied by the decision-making process, regardless of whether the individual is future-oriented. Therefore, future orientation must work together with other variables to shape green consumption [29].

### 4.2. Matching Effect of Future Orientation and Construal Level

The construal level was closely related to time perspective; Hypothesis 2 was confirmed, which stated that high magnitudes of future orientation and the construal level predict the greatest willingness to not only consume green products and green consumption behaviors but also purchase environmentally friendly products.

An abstract construal level will have a gainful effect on the future-oriented time perspective to promote green consumption. Given a long-term time orientation, individuals can demonstrate greater self-control and think about the long-term benefits of green consumption [23]. And with an abstract construal level, one can easily make meaning for future investment [48], for example, protecting the environment, thus demonstrating an enhancement effect of future orientation and the construal level [31]. When an individual’s construal level is more specific, although the individual’s temporal perspective may focus on future events, when confronted with a green consumption situation, the individual’s focus is more likely to be on the details or feasibility of environmentally friendly products or behaviors in the future. Hence, they may make decisions that are detrimental to the environment because environmentally friendly products are typically more expensive, or conventional products may be more appealing and comfortable [33]. Therefore, when it is hoped to increase green consumption by enhancing an individual’s future orientation, this should be accompanied by the activation of abstract construal so that future orientation and its enhancement effect can play a joint role [34].

### 4.3. Theoretical Contribution and Practical Implications

Theoretically, in terms of future orientation and the construal level, this study extends theories related to explaining green consumption. First, this study creatively proposed and verified an enhancement effect of future orientation and the construal level based on time perspective theory [20], construal level theory [31], and the enhancement effect of time fit [34]. Higher levels of green consumption can only be predicted when future orientations and the abstract construal level are combined. This explains the inconsistent relationship between future orientation and green consumption in previous studies and identifies marginal conditions for future orientation to promote green consumption. Second, research in the field of green consumption suffers from poor measurement validity, in which the separation of attitudes and actions can lead to research bias [18]. This study introduced the dual validation of self-report scales and behavioral choice paradigms, providing a paradigmatic basis for research in this area.

Practically, the findings provide guidance and recommendations for future interventions and environmental advocacy. First, future orientation interventions to promoting green consumption can be developed, drawing on future-oriented interventions in the areas of psychotherapy and education [45,46,47]. Second, future-orientation interventions should be coupled with time-perspective interventions to achieve enhanced roles in the field. When advertising green products, adverts should not only contain information that leads individuals to think about the future but also include high construal-level information that integrates future consequences and personal responsibility to prevent individuals from paying too much attention to detailed information about non-environmentally friendly products and making decisions based on personal preferences. Third, the findings provide practical guidance for enhancing the precision and personalization of green consumption interventions. For individuals with low future orientation, it is necessary to provide a coordinated intervention of future orientation and the construal level to enhance the intervention effect. For individuals with high future orientation, simply adopting a self-distancing intervention and changing the individual’s construal level can save intervention resources while achieving intervention effects [49].

## 5. Conclusions

Future orientation positively predicted green consumption intentions and behaviors, while the construal level played a moderating role. High construct levels and high future orientations are best matched when it comes to enhancing green consumption intentions and behaviors. This study has some limitations. First, that the sample was obtained through convenience sampling limits the generalizability of the results; these issues can be tested in future studies with larger sample sizes. Second, while this study found that trait future orientation and the construal level interacted to influence green consumption one month later, given that future orientation and the construal level also include a state component, future research can explore priming individuals with different temporal perspectives and construal levels to observe whether there remains an augmenting effect of stateful change on future orientation and the construal level. Third, how to cross the intention–behavior gap in the field of green consumption is an important social concern, and while consistent results were obtained from green consumption intentions and behaviors in this study, there is still a need for future research to explore how to promote green consumption behaviors from green consumption intentions [16]. Finally, although we incorporated an actual product purchase paradigm, this decision also does not fully reflect the actual behavior of individuals in the green consumption process. And because future orientation and the construal level are idiosyncratic variables that can change in everyday life, future research can explore the enhancement effects of future orientation and the construal level in more ecologically valid ways, such as using intensive tracking or ecological momentary assessment to verify whether the effects are stable, and tracking the green consumption behavior in real life, for example, the family expense in green consumption compared with hedonic consumption.

## Figures and Tables

**Table 1:** Correlation analysis.

	1	2	3	4	5	6
1 Age	1					
2 Future orientation T1	0.003	1				
3 Construal level T1	0.105	0.260 **	1			
4 Green consumption intention T2	0.055	0.373 **	0.085	1		
5 Green consumption behavior T2	0.169 *	0.358 **	0.172 *	0.742 **	1	
6 Purchase decision	0.063	0.103	0.051	0.310 **	0.386 **	1

* *p* < 0.05, ** *p* < 0.01.

## Data Availability

The data that support the findings of this study are openly available in [Figshare] at https://figshare.com/s/933cb7c7f42e7cc93735, accessed on 15 November 2024.
